# Are Luxury Brand Labels and “Green” Labels Costly Signals of Social Status? An Extended Replication

**DOI:** 10.1371/journal.pone.0170216

**Published:** 2017-02-07

**Authors:** Joël Berger

**Affiliations:** 1 Utrecht University, Department of Sociology, Utrecht, The Netherlands; 2 University of Zurich, Institute of Sociology, Zurich, Switzerland; University of Exeter, UNITED KINGDOM

## Abstract

Costly signaling theory provides an explanation for why humans are willing to a pay a premium for conspicuous products such as luxury brand-labeled clothing or conspicuous environmentally friendly cars. According to the theory, the extra cost of such products is a signal of social status and wealth and leads to advantages in social interactions for the signaler. A previous study found positive evidence for the case of luxury brand labels. However, an issue of this study was that some of the experiments were not conducted in a perfectly double-blind manner. I resolved this by replicating variations of the original design in a double-blind procedure. Additionally, besides the luxury label condition, I introduced a “green” label condition. Thus, the hypothesis that signaling theory is able to explain pro-environmental behavior was tested for the first time in a natural field setting. Further, I conducted experiments in both average and below-average socioeconomic neighborhoods, where, according to signaling theory, the effects of luxury signals should be even stronger. In contrast to the original study, I did not find positive effects of the luxury brand label in any of the five experiments. Nor did I find evidence for a green-signaling effect. Moreover, in poor neighborhoods a negative tendency of the luxury label actually became evident. This suggests that a signaling theory explanation of costly labels must take into account the characteristics of the observers, e.g. their social status.

## Introduction

### Conspicuous consumption as a signal of social status

Sociologist and economist Thorstein Veblen [1] coined the term *conspicuous consumption*, referring to the display of easily recognizable expensive goods when cheaper functional equivalents exist. Examples are sports cars, expensive watches, and luxury clothes. Why are humans willing to pay a luxury premium for such items?

Costly signaling theory (CST) [2–6] offers a parsimonious explanation. CST is based on the following tenets: First, members of a group vary with respect to a desirable yet not directly observable quality. Second, this quality is correlated with the signal in a reliable way, which means that lower-quality individuals are not able or cannot afford to emit the signal. Third, an observer derives some benefit from the possibility to discriminate between individuals with different levels of this quality. Fourth, signaling allows the receiver of the signal to make an inference about the sender’s quality [7, 8].

This implies that, according to CST, an individual displaying luxury items signals that she or he is able to “waste” money and is thus wealthy and of a high social status, which is the unobservable yet desirable quality. Humans have a preference for bonding with wealthy and high-status individuals and thus treat them more favorably in social interactions [9, 10]. Since neither wealth nor social status is directly observable, when bonding, humans rely on signals for these traits [11–13]. Obviously, according to the theory, the luxury signals provide benefits to both signalers and observers.

Nelissen and Meijers chose the example of luxury brand-labeled clothes to test the CST explanation for conspicuous consumption [14]. They are the first to present evidence suggesting that individuals displaying luxury brands are indeed treated more favorably during social interactions.

### Public generosity and pro-environmental behavior as signals of status and prosociality

It has been suggested that unconditional prosocial behavior in public (e.g., public generosity) is an even more efficient investment in social capital (i.e., bonding with desirable allies) than the mere display of luxury goods. As is the case with the consumption of expensive goods, public generosity imposes costs on the signaler and is thus a signal of wealth and social status. In addition, potential allies may infer that the signaler will spread his or her benevolence to them too. Hence, the desire to associate with prosocial signalers might even be greater than the wish to affiliate with luxury signalers [15].

As an example of prosocial signaling, among the Meriam, a small-scale tribal society in the South Pacific, young men hunt turtles and present them to the community in a public feast. This gift is not reciprocated by the other group members. However, the turtle hunters gain in status; as a result, they have the opportunity to mate with one of the most desired women and have a higher number of offspring than average [16]. Similar phenomena have been reported for other tribal societies [17–20].

Analogous to turtle hunting in modern societies are non-anonymous donations [21–23]. It has been shown experimentally that individuals prefer to engage publicly rather than privately in prosocial behavior [24], and that publicly generous individuals gain in social status [25, 26] and are perceived as more cooperative and trustworthy [27–29]. Consequently, they are more often chosen as allies, economic exchange partners [30–32], and group leaders [33,34]. In some instances, they can even expect higher returns from economic transactions in the long run [35]. Probably in anticipation of such advantages, under conditions of partner selection, individuals even compete to be the most altruistic, sending a signal of prosociality and status in order to maximize their chances of securing the most desired allies. This behavioral pattern of “competitive altruism” [36] was first detected in animals [37, 38] (but see [39, 40]) but has also been observed in humans [41, 42].

More recently, it has been pointed out that conspicuous environmentally friendly behavior is an instance of conspicuous prosocial behavior and thus can be explained within the framework of CST [43, 44]. A typical example of a “green signal” is the hybrid car Toyota Prius. Compared to fuel-powered models in the same price segment it offers less comfort. This is the cost that makes the signal reliable: Only an individual who truly cares about the environment, and thus the public, is willing to forgo comfort for the greater good. This interpretation is strengthened by the fact that the Prius has no conventional counterpart. It has been argued that this explains the huge success of the Prius relative to other hybrids: The former is unique and therefore can serve as a signal, while the latter cannot easily be distinguished from their fuel-powered counterparts [45]. Indeed, recent findings corroborate this hypothesis [46–48]. Similarly, in fictive purchase situations, subjects favor the ecological product over its conventional equivalent if the ecological product is costlier and conspicuous [49]. This is consistent with the hypothesis that “green” products serve as a signal of both status and prosocial attitudes and so increase people’s attractiveness as allies and exchange partners. Indeed, in experimental games people donating to a climate fund gain in reputation [50].

In a nutshell there might be two different processes from which green signalers benefit. First, as with luxury consumption, they signal their ability to “waste” money and are thus perceived as higher in status and wealthier. Second, since they buy not merely luxury products but products benefiting the environment (and thus, the public), they are also perceived as more cooperative and trustworthy. Both mechanisms enhance their desirability as interaction partners.

### The present study

The purpose of the present study is twofold. First, I aimed to reproduce the findings by Nelissen and Meijers, which suggest that the display of luxury brand yields benefits in social interactions. In doing so, I expanded on the original design by adding a perfectly double-blind procedure, suggested by Nelissen and Meijers for further research. Second, I conducted a first test of the hypothesis that green signals have the same favorable impact on social relations as luxury brands in a natural field setting. Additionally, since I could not replicate the original findings, I conducted the experiments in neighborhoods with average and below-average socioeconomic status. This is of interest because CST predicts the effects of luxury brand labels to be stronger in neighborhoods with a below-average socioeconomic status since the costs of a luxury brand are higher for less wealthy individuals, and therefore the signal is perceived as more reliable.

The remainder of this article is structured as follows. First, I investigate how the labels exploited in the study at hand are perceived (experiment 1). Thereafter, I test whether people are more likely to comply with our confederate’s request to take part in a short survey when wearing a luxury brand-labeled shirt or a green (environmentally friendly) shirt as compared to the control condition without a label. In experiment 3, I test the hypothesis that displaying luxury labels or green labels yields financial benefits. Experiment 4 is a replication of the compliance study in a neighborhood with low socioeconomic status, while in experiment 5 money is collected in a below-average socioeconomic neighborhood. All experiments but one were conducted with both the original procedure and a new, double-blind variant.

## Experiment 1: Status Perception

As a precondition for a brand label to work as a signal it must be associated with a desirable trait. As discussed in the introduction, I expect that luxury labels are associated with higher ratings in regard to status and wealth. Green labels could also correlate with higher ratings in regard to status and wealth because green products are typically more expensive than their conventional counterparts. As an additional mechanism, they could correlate with higher ratings on the dimension ‘cooperativeness’ and ‘trustworthiness’, because green signalers are willing to take on a cost in order to benefit the environment—and thus the public. There is no reason, however, to expect that luxury brand labels will invoke higher ratings in respect of ‘cooperativeness’ or ‘trustworthiness’. Hence, I measured the perception of the same individual when wearing a luxury brand-labeled shirt, an organic-labeled shirt and a baseline shirt without a label.

### Materials and method

150 passersby (*M*_age_ = 40.0 years, S.D. = 14.83, 59.33% female) where surveyed at several spots in the inner city of Zurich. Participants were randomly assigned to one of three conditions, namely no label (*n* = 50), luxury label (*n* = 50), and green label (*n* = 50). Lacoste is the better known of the two luxury brands used in the original study; thus I used Lacoste as a luxury brand label. For the green-signaling condition, I used the *Bio* label. Bio is a well-known Swiss label indicating organic products that are more expensive than equivalent non-organic products, and so fulfills a precondition for working as a costly signal. In the original study, besides ‘no label’ a low-cost brand label was also used as a control. However, no difference between either controls was detected and thus I restrict ourselves to ‘no label’ here. The photographs used in experiment 1 can be found in S1 File.

Respondents were invited to take part in a short study on the formation of first impressions and to spontaneously answer the questions without further reflection. The questionnaire consisted of a photograph of a female confederate wearing a polo shirt on which one of the two above-mentioned labels was digitally superimposed, or a polo shirt without a label. Participants rated on a scale from 1 (not at all) to 5 (completely) the person’s status (this person has a high status), wealth (this person has money), attractiveness (this person is attractive), kindness (this person is kind), trustworthiness (this person is trustworthy), prosociality (this person is prosocial), and environmental consciousness (this person is environmentally conscious). For each dependent variable (i.e., each rating), tests of significance were conducted by means of an OLS regression model, with both labels (luxury and green) as dummy variables and the control condition as a reference category. Cluster-robust standard errors were used because an individual’s ratings for the traits are not statistically independent. In addition, I report Cohen’s d as a measure of effect size.

The data generated in experiments 1–5 is available online (S2 and S3 Files).

### Ethics statement

The five conducted studies are observational field experiments. Given the nature of the studies, informed consent was not possible. However subjects were all people in public spaces (where displayed behavior is generally visible to others). I also did not record any personal identifying information. The ETH Zurich Ethics Commission approved all field experiments and waived the need for written informant consent from the participants. The person in the picture (S1 File) is an undergraduate research assistant. She has given written informed consent, as outlined in the PLOS consent form, to publication of her photograph.

### Results

Table 1 summarizes the results. In line with CTS and the original findings, the individual is perceived as higher status when wearing the shirt with the Lacoste brand label, (*t* = 1.84, *p* = .068, *d* = .368) and, more clearly, as wealthier (*t* = 2.02, *p* = .045, *d* = .406) when compared to the control condition. Interestingly, the Lacoste label *lowers* the ratings on the dimensions “trustworthiness” (*t* = -1.68, *p* = .096, *d* = -.335), “prosociality” (*t* = -2.87, *p* = .005, *d* = -.584, not measured in the original study) and “environmental conciousness” (*t* = -1.90, *p* = .059, *d* = -.382), while it does not influence perception of “attractiveness” (*t* = 0.37, *p* = .709, *d* = .075) and “kindness” (*t* = .-39, *p* = .699, *d* = -.078). Evidently, even though people wearing luxury brand labels are perceived as having a higher social status they are not necessarily seen as being more trustworthy and prosocial. In fact, the opposite could even be the case. It seems as if there could be a trade-off between displays of wealth on the one hand and perceived prosociality and trustworthiness on the other. The only clearly statistically significant and strong effect of the green label is a substantially higher average rating on the environmentally consciousness dimension (*t* = 3.82, *p* = .000, *d* = .776). Obviously, the higher rating on the pro-environment dimension was not transferred to other dimensions, such as prosociality. Also, even though Bio products are more expensive, the person in the picture was not perceived as richer in the green condition compared to the control condition. This might be due to the fact that pro-environmental attitudes are generally not associated with wealth in the public perception.

**Table 1:** Perception ratings (experiment 1).

	Status	Wealth	Attractiveness	Kindness	Trustworthiness	Prosociality	Environmental conscious
Luxury	0.320 +	0.286*	0.080	-0.060	-0.340+	-0.460**	-0.393+
label	(1.84)	(2.02)	(0.37)	(-0.39)	(-1.68)	(-2.87)	(-1.90)
	[.368]	[.406]	[.075]	[-.078]	[-.335]	[-.584]	[-.382]
Green	0.120	-0.214	0.240	0.080	0.020	-0.079	0.776***
label	(0.70)	(-1.51)	(1.28)	(0.61)	(0.11)	(-0.47)	(3.82)
	[.138]	[-.308]	[.257]	[.122]	[.023]	[-.095]	[.779]
Constant	3.400***	2.714***	3.360***	4.280***	3.940***	3.939***	3.653***
	(26.61)	(29.43)	(24.13)	(47.28)	(30.52)	(36.89)	(25.42)
*N*	150	147	150	150	150	147	148

*Notes*: A confederate approached passersby in the city and asked them to rate a person on a photograph (another confederate) on a scale from 1 to 5 with respect to status, wealth, attractiveness, kindness, trustworthiness, prosociality, and environmental consciousness when wearing a shirt with no label (control conditions), a Lacoste label (luxury label condition) or a Bio label (green label condition). The tables list coefficients from OLS regression models (*t* statistics in parentheses;

^+^ < 0.10,

* p < 0.05,

** p < 0.01,

*** p < 0.001; cluster-robust standard errors).

“No label” is the reference category. Additionally, Cohen’s *d* as a measure of effect size is reported in brackets.

## Experiment 2: Compliance

To test whether individuals wearing brand-labeled shirts are not only perceived differently but also treated more favorably in social interactions, I conducted experiments 2 and 3. Specifically, in experiment 2 I test the hypothesis that passersby more often comply with a request of a luxury signaler and a green signaler because they are perceived as higher status.

### Materials and method

720 passersby (*M*_age_ = 36.4 years, S.D. = 11.8, 61.11% female) were approached by a female confederate (as in the original study by Nelissen and Meijers) in summer 2013 at different spots in the inner city of Zurich. Following the procedure of the original study, the confederate approached with a questionnaire and clipboard and asked the targeted individual to answer a few short questions. Only unaccompanied individuals were chosen. In experiment 1 as in all other experiments, the age and gender of the participants were estimated by the respective confederates. In order to exclude a selection-bias, the confederate applied the following rule. The first person who passed the location alone was defined as the first subject. When the confederate had finished with this subject, the next person who passed the location alone was the next subject and so on. The confederates wore new blue jeans and clean sneakers in all of the experiments.

There were three experimental conditions and two different experimental procedures. Under the standard procedure, as in the original study, the confederate wore a polo shirt without a label (*n* = 120), a polo shirt with a luxury brand label (*n* = 120), or a shirt with a green label (*n* = 120). For experiment 2 and for all other experiments I used the labels introduced in the section on experiment 1. Under the alternative procedure, instead of shirts, caps with the respective labels were used (no label *n* = 111, luxury label *n* = 129, green label *n* = 120). The cap procedure was suggested by Nelissen and Meijers for future studies in order to eliminate potential experimenter effects in their own experiments. In the original procedure with polo shirts, experimenter effects cannot be ruled out even if the confederate is blind to the hypothesis under study since the treatment might be obvious. However, in the present study, the confederate was not aware of which cap she wore because she drew it blindly from a bag and put it back before drawing anew. This procedure, of course, leads to a somewhat uneven distribution of cases between the treatments of each cap experiment. The author supervised the confederate every day at varying points in time to ensure that this double-blind procedure was applied properly.

Importantly, the same confederate conducted all trials of experiment 2. Moreover, the shirts as well as the caps were identical except for the labels that were taken from original items (photographs of all items are presented in S1 File). In order to rule out effects of potential time-dependent confounding effects, the confederate chose the first experimental condition randomly and then repeatedly changed the cap or shirt after a predefined number of trials. However, due to practical reasons, the cap and shirt trials were conducted on different days. Pictures of the prepared shirts and caps used in experiments 2–5 are presented in S1 File.

### Results

Statistical tests were conducted with Fisher’s exact test. Compliance (yes = 1, 0 else) is the dependent variable and the two comparisons of interest are luxury label vs. control and green label vs. control in a first step. The shirt and cap data was analyzed separately. In a second step, the same tests were conducted for the pooled shirt and cap data. In all of the subsequent experiments, I used this strategy for data analysis (except for experiment 5, where only the shirt conditions were implemented. The related cross tabulations and test statistics are given in S5 File.

**Fig 1:**
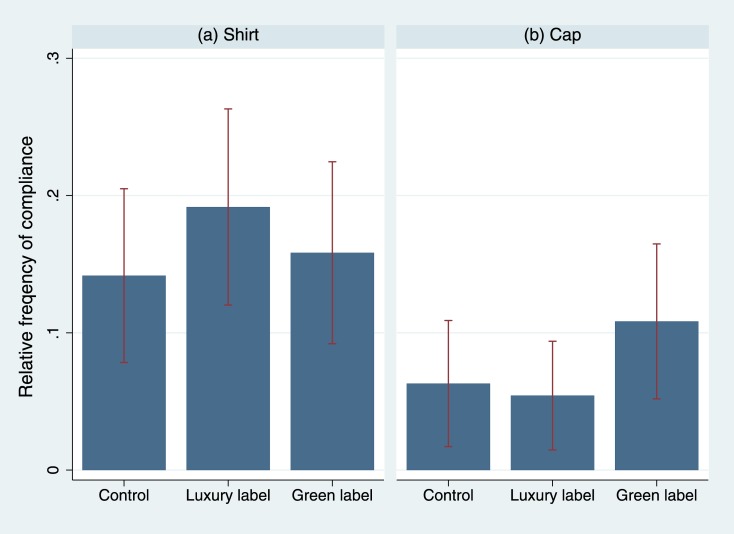
Relative frequency of compliance by experimental condition in experiment 2. A confederate approached passersby in the city and asked them to take part in a short survey. Experimental conditions: No label (control condition), Lacoste label (luxury label condition), Bio label (green label condition), printed on shirts or caps (double-blind procedure). 95% ci. The corresponding statistics can be found in S5 File.

Fig 1 summarizes the results. No statistical difference between the control condition and the luxury condition becomes evident, either in the case of shirts (*p* = .387, φ = .067) or in the case of caps (*p* = .789, φ = -.019; pooled shirt/cap data: *p* = .665, φ = .026). The same holds true for the green label (shirt: *p* = .857, φ = .131; cap: *p* = .249, φ = .080; pooled: *p* = .393, φ = .046). Contrarily, passersby complied with a significantly lower probability with the request when the confederate wore a cap (*p* = .000, φ = -0.137), independent of the label. Evidently, the type of clothing worn was more important for the perception of a person than labels.

## Experiment 3: Charity Donations

While the former experiment was designed to investigate whether people are more likely to comply with a request stemming from an individual wearing a luxury brand label or a green label, the third experiment investigates whether wearing labeled clothes yields financial benefits.

Again, this might be the case because signalers are perceived as higher status and thus observers comply with their request. For green labels, a second process might reinforce this tendency. Green signalers might be perceived as more cooperative and trustworthy, which might enhance the chances that people donate, believing that their donations would indeed reach the target instead of being misused.

For this study, I cooperated with Kinderkrebshilfe, a well-known charity that supports children suffering from cancer as well as their parents (e.g., by providing counseling and financial aid).

### Materials and method

A female confederate (as in the original study) who had not taken part in experiment 1 approached 880 passersby (*M*_age_ = 41.7 years, S.D. = 13.6, 61.7% female) at different spots in the city of Zurich. The locations were allocated by the city to charity campaigns.

The confederate approached passersby with a moneybox and information on the charity. She explained that she was collecting money for Kinderkrebshilfe and handed out literature. The confederate wore the same shirts or caps as those used in experiment 2. Each time after receiving a donation, the confederate put the money in a treatment-specific envelope. The dependent measure was the average amount of money collected.

### Results

T-tests for mean differences between average donations and Fisher’s exact test for differences in the passersby’s propensity to stop and listen to our experimenter’s request were used (see S5 File). While the first measure was reported in the original study, I additionally report the second because signals and cues could also affect a person’s propensity to give, instead of the amount he or she gives [51]. The average donation per treatment and the proportion of passersby who stopped to listen to our confederate’s request are listed in the S5 File.

**Fig 2:**
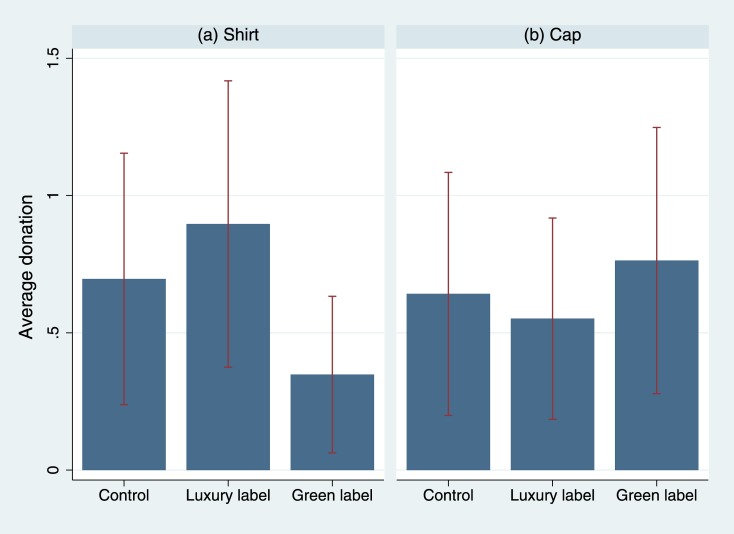
Average donation by experimental condition (experiment 3). A confederate approached passersby in the city and asked them to donate to a charity. Experimental conditions: No label (control condition), Lacoste label (luxury label condition), Bio label (green label condition), printed on shirts or caps (double-blind procedure). 95% ci. The corresponding statistics can be found in S5 File.

As evident from Fig 2, neither the Lacoste label (shirt: *t* = -.569, *p* = .570, *d* = -.064; cap: *t* = .306, *p* = .760, *d* = .038; pooled: *t* = -.312, *p* = .749, *d* = -.027) nor the Bio label (shirt: *t* = 1.275, *p* = .203, *d* = .143; cap: *t* = -.366, *p* = .715, *d* = -.044; pooled: *t* = .619, *p* = .536, *d* = .051) provided any benefits when collecting money for charity. In contrast to the former experiment, no difference between the collected amounts of money when wearing a cap and wearing a shirt is observed (*t* = .047, *p* = .962, *d* = .003) A negative effect of wearing a cap becomes evident, however, when analyzing the relative frequency of people who stop to listen to our confederate’s request (*p* = .000, *φ* = -0.206), while again, the labels do not impact the frequency of people who stop for shirts (luxury: *p* = .457, *φ* = -.049; green: *p* = .325, *φ* = -.062) or caps (luxury: *p* = .538, *φ* = .040; green: *p* = .336, *φ* = .065; pooled, luxury: *p* = 1.000, *φ* = .001; green: *p* = 1.000, *φ* = .001).

## Experiment 4: Compliance in a Neighborhood with Low Socioeconomic Status

In contrast to the original study, and in spite of the considerably larger number of subjects, a positive effect of labels on either compliance or on charitable donations could not be detected. Nor could I find a positive effect of the green label. This holds true for both shirts and caps.

A precondition for brands to function as a signal is that they are perceived as costly. Although study 1 demonstrates that this condition is met at least for the Lacoste label, I replicated the compliance experiment in a neighborhood with a low socioeconomic status in Zurich since this should raise the relative cost of the brands from the perspective of people on a low income.

### Materials and method

720 subjects were approached (*M*_age_ = 45.0, S.D. = 14.9, 58.9% female), either by the female confederate (shirt experiment) from experiment 4 or by a new, male confederate (cap experiment), and asked if they were ready to take part in a short survey (see the section on experiment 2 for further details). A male confederate was introduced in addition to the female confederate, because luxury signals might work differently depending on the gender of the signaler and of the observer [52, 53], although Nelissen and Meijers did find beneficial effects of luxury brands for both women and men.

While the shirt experiment was conducted at a street corner in the city center, the cap experiments was conducted with people waiting at a bus stop. This implies that the baseline cooperation cannot be directly compared between the shirt and the cap procedure. However, if CTS applies, the postulated effects should be observable under both procedures.

### Results

**Fig 3:**
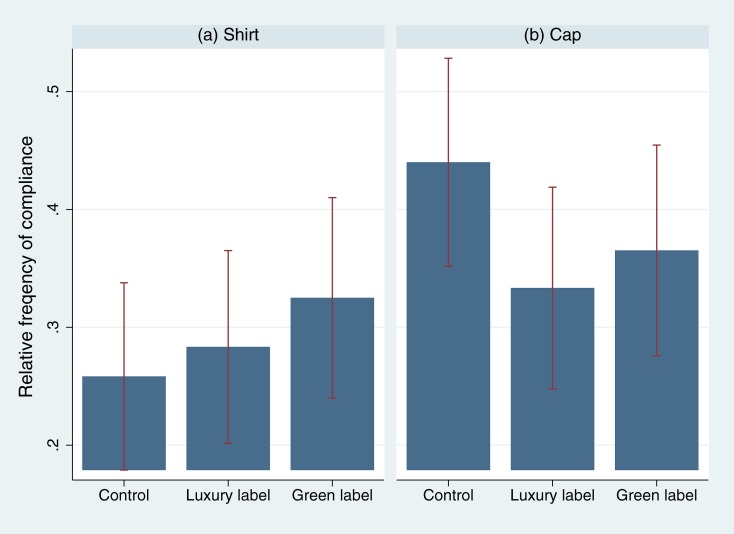
Relative frequency of compliance by experimental condition in experiment 4. A confederate approached passersby in the city (neighborhood with low socioeconomic status) and asked them to take part in a short survey. No label (control condition), Lacoste label (luxury label condition), Bio label (green label condition), printed on shirts or caps (double-blind procedure). 95% ci. The corresponding statistics can be found in S5 File.

As in study 2, neither the luxury label (shirt: *p* = .772, *φ* = .028; cap: *p* = .090, *φ* = -0.109; pooled: *p* = .335, *φ* = -.045) nor the green label (shirt: *p* = .320, *φ* = .073; cap: *p* = .292, *φ* = -.076; pooled: *p* = .924, *φ* = -.007) positively affected the rate of compliance (see Fig 3 for an overview. S5 File provides frequencies for all treatments). In fact, there is even a negative tendency for the luxury branded cap—but not for the luxury branded shirt—at a 10% level of significance. The result that this time the baseline compliance is higher in the cap experiment (*p* = .011, *φ* = .097) is most probably due to the fact that the cap experiment was conducted with participants waiting at a bus stop, while in the shirt experiment, passersby were approached. All the same, in either case labels have no positive effect on compliance.

## Experiment 5: Charity Donations in a Neighborhood with Low Socioeconomic Status

In study 4, which was conducted in a neighborhood with low socioeconomic status, for the first time an effect at least reached the 10% significance level (even though in the opposite direction than hypothesized). For this reason, I also replicated study 3 (i.e., collecting money for a charity) in a neighborhood with below-average socioeconomic status. Instead of Zurich the experiment was conducted in the Swiss capital Bern to ensure that it is not an unknown idiosyncratic trait of the inhabitants of Zurich that is responsible for the lacking effect of the brand labels.

### Materials and method

The same male confederate as in experiment 4 approached 360 individuals near a shopping center in a neighborhood with a low socioeconomic status (*M*_age_ = 48.9, *S*.*D*. = 13.8, 53.3% female). The procedure was equivalent to the one of experiment 3, except that only the shirt procedure was realized. The beneficiary of the collected money was the same charity as before.

### Results

**Fig 4:**
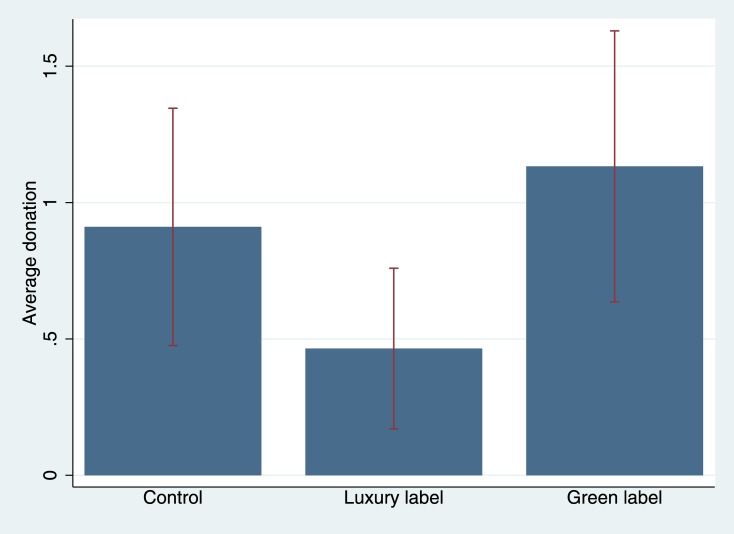
Average donation by experimental condition (experiment 5). A confederate approached passersby in the city (neighborhood with low socioeconomic status) and asked them to donate to a charity. Experimental conditions: No label (control condition), Lacoste label (luxury label condition), Bio label (green label condition), printed on shirts. 95% ci. The corresponding statistics can be found in S5 File.

Fig 4 displays the main results. The average donation per treatment and the proportion of passersby who stopped to listen to our confederate’s request are listed in the S5 File. In comparison to the shirt without a label, the luxury labeled shirt negatively impacts the average amount of money collected (*t* = 1.683, *p* = .094, *d* = .217), while the green label has no effect (*t* = -.665, *p* = .507, *d* = -.086)–a result that is consistent with the findings of study 4, where the luxury label negatively affected compliance.

A similar tendency (which is, however, not statistically significant) becomes evident when looking at the passersby’s propensity to stop (control vs. luxury: *p* = .478, *φ* = -.055, control vs. green: *p* = 1.000, *φ* = .000).

## Robustness Analyses

Our main finding is that individuals displaying luxury brand labeled clothes or green labeled clothes are not treated more favorably in social interactions. Rather, luxury labeled clothing even seems to have a negative impact in low socio-economic status neighborhoods. In what follows I address the robustness of these findings. To be more precise, I discuss whether a lack of statistical power or gender interaction effects could explain the absence of positive treatment effects.

### Statistical power and pooled analyses

Even though in each of the studies the sample size is considerably larger than in the original study, there is a risk, which cannot be ignored, of a type II error being present in some of our studies, when assuming the treatment effects to be considerably lower than those reported in the original study. In order to rule out the possibility that the null effects can exclusively be attributed to low statistical power, I provide power analyses of all the studies in S6 File.

It becomes evident that in some studies there is indeed a non-negligible risk of type II error (statistical power below 80%) present—at least when assuming the actual treatment effects to be considerably lower than the ones reported in the original study. However, in most of the studies a sufficient level of statistical power is reached. Still, I fail to replicate the positive treatment effects found in the original study.

More specifically, power is more of an issue in the charity donation studies than in the survey (compliance) studies. However, in the first charity donation study (study 3), the effect of the luxury brands points in different directions for shirts and for caps. As a result, even when pooling the shirt and the cap data (reaching a power of over .8), there are no significant treatment effects. In the donation study in the low status neighborhood (study 5) I actually find a negative effect of the luxury brand (despite the rather low power).

To further corroborate our results, I pooled the data, increasing statistical power. That is, I generated the variable ‘positive reaction’, which is 1 if a person took part in the brief survey (studies 2 and 4) or stopped to listen to the confederate’s request (studies 3 and 5), and is 0 otherwise. I conducted separate analyses for neighborhoods with average and low socioeconomic status because our results suggest that the treatments could have different effects in both neighborhood types. However, in the pooled data I also do not find any positive treatment effects. In addition, I replicated the analyses with logit models that contain study-specific dummy variables in order to control for between-study variance. However, this also did not change the results (S8 File). In sum this suggests that the absence of positive treatment effects cannot be attributed to a lack of statistical power.

### Gender-specific interaction effects

Another explanation for the many null effects would be that observers react differently towards the labels depending on the gender of the experimenter and their own gender (gender-specific interaction effects). Specifically, theoretical arguments and empirical evidence suggest that within-gender competition could lead to a negative treatment of status-seeking signalers by observers of the same gender—particularly for males [52, 53]. For example, while female subjects would react positively to a status-signaling male confederate, male subjects would punish status-signaling male confederates by refusing to participate in a survey or to donate any money. This behavior of the male subjects would then reduce a positive treatment effect at the aggregate level.

In order to achieve a reasonable amount of observations per cell, I pooled the data of the two compliance studies (studies 2 and 4) as well as the data of the two donation studies (studies 3 and 5). Additionally, I pooled the data of all four studies. The results of the gender-specific analyses are tabulated in the S7 File.

As before, I do not find a significantly positive effect for any label. However, I find that the negative effect of the luxury brand is predominantly driven by male subjects. Male subjects have a lower tendency to comply with the request to take part in a short survey (although not statistically significantly so) or to give a donation to charity (p = .028) when the male confederate is wearing a luxury brand label, as compared to the control condition. This supports the notion of within-sex competition among males. However, this single effect cannot explain the absence of positive treatment effect at the aggregate level since in mixed-gender constellations there are also no clear positive treatment effects.

## Discussion and Conclusion

Nelissen and Meijers argue that CST explains the human taste for luxury goods in general and for luxury brand labels in particular. According to the theory, an individual displaying luxury items signals that she or he can afford to “waste” money and is thus wealthy and of a high social status. This leads to an advantageous treatment of the signaler in social interactions because humans have a preference for associating with wealthy and high-status people and are ready to pay a premium to affiliate with them. Because neither wealth nor social status is directly observable, when bonding, humans rely on signals, inferring these traits.

As an extension of the luxury-signaling thesis it has been suggested that public prosociality, such as conspicuous generosity or environmentally friendly behavior, works in an analogous way. Public prosociality may function as a signal of wealth and, additionally, of prosocial values and cooperativeness. Hence, people sending prosocial or pro-environmental signals, like people sending luxury signals, should be treated advantageously in social interactions.

The present study aimed to replicate the original study by Nelissen and Meijers. Expanding on their design, I implemented a perfectly double-blind procedure proposed by the authors of the original study and, additionally, a green-signaling condition. The latter provides a first test of the hypothesis that conspicuous pro-environmental behavior works in an analogous way to conspicuous consumption in a natural field setting. Further, I conducted experiments in neighborhoods of average as well as below-average social status. In the latter, effects of costly labels are predicted to be even stronger since the signal should be perceived as more reliable from the perspective of less wealthy observers.

While I replicate the finding of the original study of an individual wearing a luxury brand-labeled shirt being rated as higher status and wealthier than an individual wearing a shirt without a label, in contrast to the original study, the individual was also perceived as less trustworthy, less prosocial and less environmentally friendly when wearing a luxury brand-labeled shirt. In the green label condition, even though the wearer was perceived as more environmentally friendly, there were no spillover effects from this to other unobservable but positive traits, such as prosociality, trustworthiness, or wealth, as predicted by the green-signaling hypothesis (experiment 1).

Moreover, neither the confederate asking people to take part in a survey (experiment 2) nor the confederate collecting money for a charity (experiment 3) was treated more favorably when wearing a luxury brand-labeled shirt or cap. Nor did the green label have an effect in experiment 2 or 3. However, wearing a cap was disadvantageous in both experiments, independent of the brand.

Subsequently, I replicated experiments 2 and 3 in two neighborhoods of low socioeconomic status in order to make sure that the cost condition of a CTS explanation was met. In both studies, no positive effect of any label became evident. On the contrary, in studies 4 (cap) and 5 (shirt), a negative tendency of the luxury brand label was observed.

I then considered different explanations for these findings. One potential explanation is a lack of statistical power. However, even when I assume positive effects to be considerably lower than those found in the original study, I achieve substantial statistical power in many of the studies. Also, when pooling all the behavioral studies (2–4), I cannot detect any positive statistically significant effects.

Another explanation would be that within-gender competition reduces treatment effects. However, gender-specific analyses do not support this explanation.

The finding that luxury labels can even lead to disadvantages for the displayer suggests that a signaling theory of brand labels must be formulated in a more finely-tuned manner. Such a theory would postulate that labels are costly signals in the sense of CST, but that being wealthy is not always perceived as positive. On the one hand, there is no general aversion towards displaying wealth in Switzerland. Specifically in Zurich, where a considerable proportion of the population works in the financial sector, displaying wealth is common. On the other hand, the negative effect of the luxury brand label in neighborhoods with a low socioeconomic status could be explained by aversion towards disadvantageous inequality [54,55]. More generally, Nelissen and Meijers’ premise that people have a preference for bonding with high-status individuals in general might be wrong. Rather, people might prefer to interact and bond with people of a similar social status. Such tendencies of homophily are not only predicted by biological market theory [56], they have also been observed not only in humans [57] but also in other primates [58]. This implies that, depending on the preferences that are dominant in a given subgroup, positive or negative features might be associated with a given brand. On average, such effects might cancel each other out, resulting in zero effect.

A tendency towards homophily could also explain our finding that wearing a cap lowers the likelihood of cooperation. Caps are worn more often by teenagers than by the average Swiss. Hence, wearing a cap might be perceived as a signal; the wearers may pay a cost in the sense that they lower their likelihood of bonding with average people, but at the same time they enhance their likelihood of associating with members of certain subgroups (e.g., younger people).

Another explanation for the lack of positive effects might be that a brand label is simply not costly enough. A low-status signaler could fake the signal in that he owns one expensive shirt, while owning a wardrobe of expensive items is a more reliable signal. Put differently, wearing one luxury item does not contain much information to discriminate between the wealthy and less wealthy. A signaling theory of luxury items should account for this point. Correspondingly, in future studies, a whole variety of clothes and accessories should be used (for example, costly watches and shoes in addition to brands). Future research should also test the homophily hypothesis of signaling. For instance, labels with a specific connotation could be tested among different subgroups. Luxury brand experiments could be conducted in neighborhoods with high socioeconomic status, with average or even low socioeconomic status neighborhoods as a control group. Accordingly, green brands could be tested in neighborhoods where people with a high level of ecological awareness.

## Supporting Information

S1 File T-Shirts and caps used in field experiments. (PDF)

S2 File Data of experiment 1. (TXT)

S3 File Data of experiments 2–5. (TXT)

S4 File Codebook. (PDF)

S5 File Cross tables and test statistics. (PDF)

S6 File Power analyses. (PDF)

S7 File Gender interaction effects. (PDF)

S8 File Logit regression models with and without study-specific dummy variables. (PDF)
