# Supplementary material for: Are Luxury Brand Labels and “Green” Labels Costly Signals of Social Status? An Extended Replication
Source: PLoS One. 2017 Feb 7;12(2):e0170216. doi: 10.1371/journal.pone.0170216 (PMC5295666; doi:10.1371/journal.pone.0170216)
Supplement: S1 File — (PDF) [file pone.0170216.s001.pdf]

S1 File: Shirts and caps used as experimental treatments

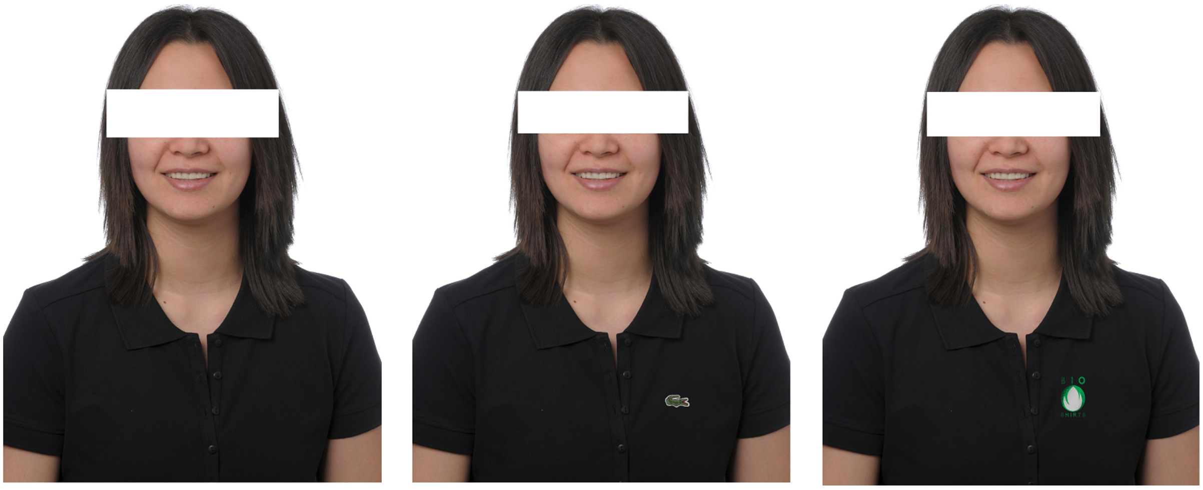

Pictures used for perception rating in experiment 1. (a) Control condition, (b) luxury label condition, (c) green label condition. The photograph was not blackened in the study but only for the purpose of anonymity in this publication.

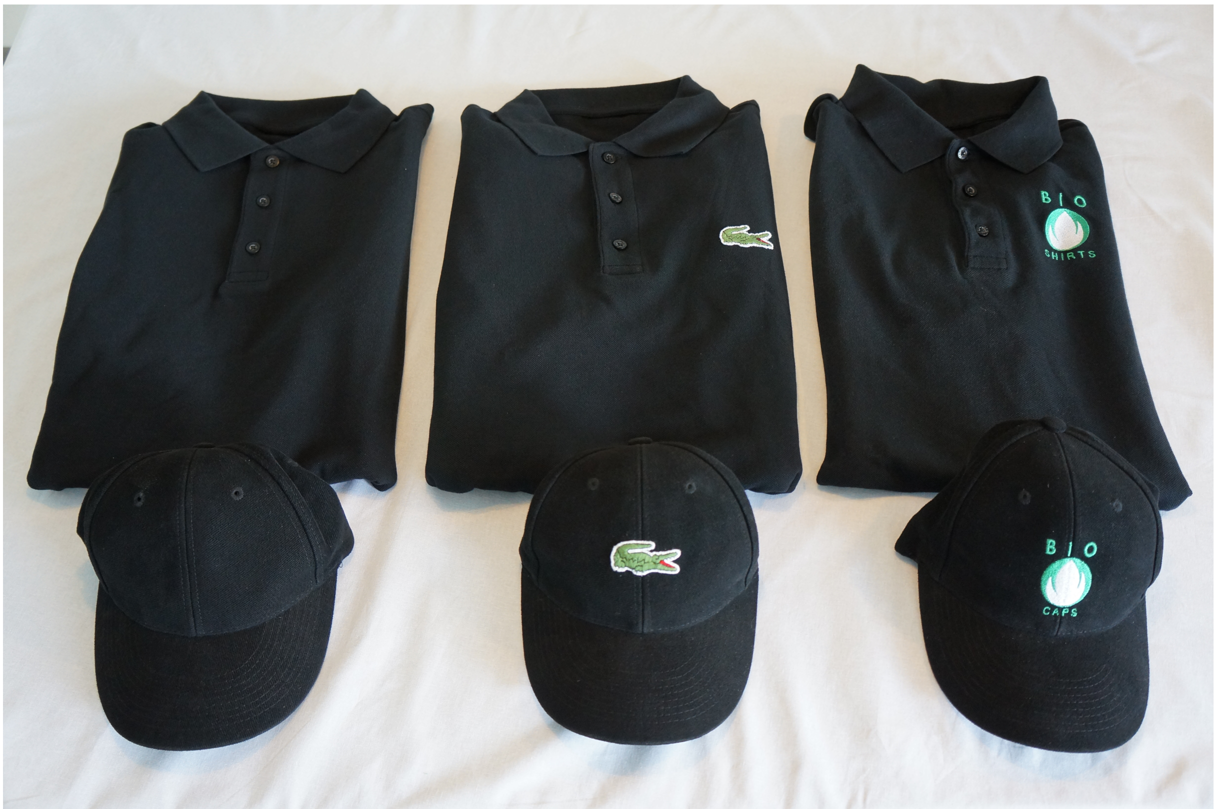

Shirts and caps used in experiments 2-5. (a) Control condition, (b) luxury label condition, (c) green label condition.
