# Supplementary material for: Are Luxury Brand Labels and “Green” Labels Costly Signals of Social Status? An Extended Replication
Source: PLoS One. 2017 Feb 7;12(2):e0170216. doi: 10.1371/journal.pone.0170216 (PMC5295666; doi:10.1371/journal.pone.0170216)
Supplement: S4 File — (PDF) [file pone.0170216.s004.pdf]

S4 File: Codebook

Experiment 1

Corresponding data: S2_File.txt

Variable	Variable description	Values	Value description
id	Subject identifier	1...N	
treat	Treatment	0, 1, 2	0: Control<br>1: Luxury (Lacoste)<br>2: Green (Bio)
status	Status	1...5	1 (low status)<br>...<br>5 (high status)
money	Wealth	1...5	1 (not wealth)<br>...<br>5 (very wealthy)
ecofriendly	Environmental consciousness	1...5	1 (no environmentally conscious)<br>...<br>5 (very environmentally conscious
trustworthy	Trustworthiness	1...5	1 (not trustworthy)<br>...<br>5 (very trustworthy)
social	Prosociality	1...5	1 (not prosocial)<br>...<br>5 (very prosocial)
attractive	Attractiveness	1...5	1 (not attractive)<br>...<br>5 (very attractive)
kind	Kindness	1...5	1 (not kind)<br>...<br>5 (very kind)
age	Age in years	1...k	
female	Female	0, 1	0: no<br>1: yes

Experiments 2-5

Corresponding data: S3_File.txt

Variable	Variable description	Values	Value description
vpnr	Subject identifier	1...N	
treat	Treatment	0, 1, 2	0: Control<br>1: Luxury (Lacoste)<br>2: Green (Bio)
female	Subject is female	0, 1	0: No<br>1: Yes
femalexp	Experimenter is female	0, 1	0: No<br>1: Yes
age	Age in years	1...k	
shirt	Shirt procedure (vs. cap procedure)	0, 1	0: No<br>1: Yes
sum	Donation in CHF (Swiss francs)	0...k, 999	999 is missing value (survey studies)
stop	Passerby stops in order to listen to request	0, 1	0: No<br>1: Yes
positive	Positive reaction: 1 if a person took part in the survey (studies 2 and 4) or stopped to listen to the confederate's request (studies 3 and 5) and equals 0 else.	0, 1	0: No<br>1: Yes
study	Study identifier	2, 3, 4, 42, 5	2: Survey average status<br>3: Donation average status<br>4: Survey low status<br>42: Survey low status busstop<br>5: Donation low status
