# Supplementary material for: Are Luxury Brand Labels and “Green” Labels Costly Signals of Social Status? An Extended Replication
Source: PLoS One. 2017 Feb 7;12(2):e0170216. doi: 10.1371/journal.pone.0170216 (PMC5295666; doi:10.1371/journal.pone.0170216)
Supplement: S5 File — (PDF) [file pone.0170216.s005.pdf]

S5 File
Cross tables: Frequencies and test statistics

Legend. C: Control treatment, L: Luxury label treatment, G: Green label treatment, exact: Fisher's exact test

Study 2 (Compliance)

Shirt

	Control		Luxury label		Green label	
	n	%	n	%	n	%
Does not comply	103	85.83	97	80.83	101	84.17
Complies	17	14.17	23	19.17	19	15.83
Total	120	100	120	100	120	100

Overall: χ^2=1.135, p=.567
CL: χ^2=1.080, p=.299, exact=.387
CG: χ^2=.131, p=.718, exact=.857
LG: χ^2= .462, p=.497, exact=.611

Cap

	Control		Luxury label		Green label	
	n	%	n	%	n	%
Does not comply	104	93.69	122	94.57	107	89.17
Complies	7	6.31	7	5.43	13	10.83
Total	111	100	129	100	120	100

Overall: χ^2=2.950, p=.229
CL: χ^2= .084, p=.772, exact=.789
CG: χ^2= 1.494 , p=.222, exact=.249
LG: χ^2= 2.460, p=.117, exact=.161

Overall

	Control		Luxury label		Green label	
	n	%	n	%	n	%
Does not comply	207	89.61	218	87.55	206	85.83
Complies	24	10.39	31	12.45	34	14.17
Total	231	100	249	100	240	100

Overall: χ^2=.974 , p=.615
CL: χ^2= .330, p=.566, exact=.665
CG: χ^2= .974, p= .324, exact=.393
LG: χ^2= .182, p=.669, exact=.686

Shirt vs. Cap: χ^2= 13.52, p=.000, exact=.000

Study 3 (Charity donations)

Passerby stops

Shirt

	Control		Luxury label		Green label	
	n	%	n	%	n	%
Does not stop	42	26.25	49	30.63	51	31.87
Stops	118	73.75	111	69.38	109	68.12
Total	160	100	160	100	160	100

Overall: χ^2= 1.340, p= .512
CL: χ^2= .752, p=.386, exact=.457
CG: χ^2= 1.23, p=.268, exact=.325
LG: χ^2= .058, p=.809, exact=.904

Cap

	Control		Luxury label		Green label	
	n	%	n	%	n	%
Does not stop	74	53.24	61	49.19	64	46.72
Stops	65	46.76	63	50.81	73	53.28
Total	139	100	124	100	137	100

Overall: χ^2= 1.196, p= .550
CL: χ^2= .429, p= .512, exact=.538
CB: χ^2=1.174, p= .279, exact=.336
LG: χ^2= .160, p=.689, exact=.711

Overall

	Control		Luxury label		Green label	
	n	%	n	%	n	%
Does not stop	116	38.80	110	38.73	115	38.72
Stops	183	61.20	174	61.27	182	61.28
Total	299	100	284	100	297	100

Overall: χ^2= .000, p=1.0
CL: χ^2=.000, p= .987, exact= 1.000
CG: χ^2= .000, p=.985, exact= 1.000
LG: χ^2= .000, p=.998, exact=1.000

Shirt vs. cap: χ^2 = 37.386, p=.000, exact=.000

Average donation

Shirt

	Control	Luxury	Green
Mean donation	.696	.896	.348
SE	.232	.264	.144

CL: t= -.569, p=.570
CG: t= 1.275, p=.203
GL: t= 1.822, p=.069

Cap

	Control	Luxury	Green
Mean donation	.642	.552	.763
SE	.224	.185	.245

CL: t=.306, p=.760
CG: t=-.366, p=.715
GL: t=-.678, p=.499

Overall

	Control	Luxury	Green
Mean donation	.671	.746	.539
SE	.162	.169	.138

CL: t=-.312, p=.749
CG: t=.619, p=.536
GL: t= .949, p=.343

Shirt vs. cap: t= .047, p= .962

Study 4 (Compliance, low status neighborhood)

Shirt

	Control		Luxury label		Green label	
	n	%	n	%	n	%
Does not comply	89	74.17	86	71.67	81	67.50
Complies	31	25.83	34	28.33	39	32.50
Total	120	100	120	100	120	100

Overall: χ^2= 1.325, p=.516
CL: χ^2= .190, p=.663, exact=.772
CG: χ^2=1.291, p=.256, exact=.320
GL: χ^2= .492, p=.483, exact=.575

Cap

	Control		Luxury label		Green label	
	n	%	n	%	n	%
Does not comply	70	56	80	66.67	73	63.48
Complies	55	44	40	33.33	42	36.52
Total	125	100	120	100	115	100

Overall: χ^2=3.124, p=.210
CL: χ^2= 2.934, p=.087, exact=.090
CG: χ^2=1.391, p=.238, exact=.292
GL: χ^2= .263, p=.608, exact=.682

Overall

	Control		Luxury label		Green label	
	n	%	n	%	n	%
Does not comply	159	64.9	166	69.17	154	65.53
Complies	86	35.1	74	30.83	81	34.47
Total	245	100	240	100	235	100

Overall: χ^2=1.147, p=.563
CL: χ^2= .999, p= .317, exact=.335
CG: χ^2=.021, p=.884, exact=.924
GL: χ^2= .713, p=.398, exact=.434

Shirt vs. cap: 6.792, p= .009, exact=.011

Study 5 (Charity donations, low status neighborhood)

Shirt: stop

	Control		Luxury label		Green label	
	n	%	n	%	n	%
Does not stop	82	68.33	88	73.33	82	68.33
Stops	38	31.67	32	26.67	38	31.67
Total	120	100	120	100	120	100

Overall: χ^2= .952, p= .621
Cont-Lux: χ^2= .726, p= .394, exact=.478
Cont-Bio: χ^2=.000, p=1.000, exact=1.000
Bio-Lux: χ^2= .726, p= .394, exact=.478

Shirt: donations

	Control	Luxury	Green
Mean donation	.911	.465	1.133
SE	.220	.149	.251

CL: t=1.683 , p= .094
CB: t= -.665 , p=.507
BL: t= -2.290, p= .023

Pooled: average neighborhood

Shirt

	Control		Luxury label		Green label	
	n	%	n	%	n	%
Negative reaction	145	51.79	146	52.14	152	54.29
Positive reaction	135	48.21	134	47.86	128	45.71
Total	280	100	280	100	280	100

Overall: χ^2= .410, p= .814
CL: χ^2= .007, p= .933, exact=1.000
CG: χ^2=.351, p= .553, exact=.611
LG: χ^2= .611, p= .258, exact=.672

Cap

	Control		Luxury label		Green label	
	n	%	n	%	n	%
Negative reaction	178	71.20	183	72.33	171	66.54
Positive reaction	72	28.80	70	27.67	86	33.46
Total	250	100	253	100	257	100

Overall: χ^2= 2.294, p= .318
CL: χ^2= .080 , p= .778, exact=.843
CG: χ^2= 1.285, p= .257, exact=.292
LG: χ^2= 2.017, p= .156, exact=.178

Pooled

	Control		Luxury label		Green label	
	n	%	n	%	n	%
Negative reaction	323	60.94	329	61.73	323	60.15
Positive reaction	207	39.06	204	38.27	214	39.85
Total	530	100	533	100	537	100

Overall: χ^2= .280, p=.870
CL: χ^2= .069, p=.793, exact=.801
CG: χ^2=.071, p= .791, exact=.802
LG: χ^2=.280, p=.597, exact=.616

Shirt vs. cap: χ^2=52.488, p=.000, exact=.000

Pooled: low status neighborhood

Shirt

	Control		Luxury label		Green label	
	n	%	n	%	n	%
Negative reaction	82	68.33	88	73.33	82	68.33
Positive reaction	38	31.67	32	26.67	38	31.67
Total	120	100	120	100	120	100

Overall: χ^2=.952 , p= .621
CL: χ^2= 0.726, p= .394 , exact= .478
CG: χ^2= .000, p= 1.000, exact= 1.000
LG: χ^2= 0.726, p= .394, exact=.621

[Cap low status study is cap study 4]

Overall

	Control		Luxury label		Green label	
	n	%	n	%	n	%
Negative reaction	152	62.04	168	70.00	155	65.96
Positive reaction	93	37.96	72	30.00	80	34.04
Total	245	100	240	100	235	100

Overall: χ^2=3.421 , p=.181
CL: χ^2= 3.421 , p= .064, exact=.069
CG: χ^2=.798, p= .372, exact=.393
LG: χ^2=0.892, p=.345, exact=.376

Shirt vs. cap: χ2= 7.912, p=.005

Pooled: all

Shirt

	Control		Luxury label		Green label	
	n	%	n	%	n	%
Negative reaction	316	60.77	320	61.54	315	60.58
Positive reaction	204	39.23	200	38.46	205	39.42
Total	520	100	520	100	520	100

Overall: χ^2= .113, p = .945
CL: χ^2= .065, p = .799, exact =.849
CG: χ^2=.004, p = .949, exact=1.000
LG: χ^2= .101, p = 751., exact=.799

Cap

	Control		Luxury label		Green label	
	n	%	n	%	n	%
Negative reaction	248	66.13	263	70.51	244	65.59
Positive reaction	127	33.87	110	29.49	128	34.14
Total	375	100	373	100	372	100

Overall: χ^2=2.469, p = .291
CL: χ^2=1.654, p = .198, exact=.209
CG: χ^2 = .024, p = .876, exact=.878
LG: χ^2= .039, p = .843, exact=.895

Overall

	Control		Luxury label		Green label	
	n	%	n	%	n	%
Negative reaction	564	63.02	583	65.29	559	62.67
Positive reaction	331	36.98	310	34.71	333	37.33
Total	895	100	893	100	892	100

Overall: χ^2=1.559, p=.459
CL: χ^2= 1.001, p =.317 , exact =.324
CG: χ^2=.023, p=.879 , exact=.883
LG: χ^2=1.327 , p=.249 , exact=.257

Shirt vs. cap χ^2= 98.555, p=.000, exact=.000
