# Supplementary material for: Are Luxury Brand Labels and “Green” Labels Costly Signals of Social Status? An Extended Replication
Source: PLoS One. 2017 Feb 7;12(2):e0170216. doi: 10.1371/journal.pone.0170216 (PMC5295666; doi:10.1371/journal.pone.0170216)
Supplement: S6 File — (PDF) [file pone.0170216.s006.pdf]

S6 File
Power Analyses

Statistical power was determined using the program ‘G*Power’ (http://www.gpower.hhu.de).
All the values refer to two-sided tests.

Study 2 (Compliance)

Statistical power (1-beta) for Fisher’s exact test

	Delta 40	Delta 20	Delta 10
Shirt (14% baseline, n_i=120)	1.000	.945	.441
Cap (6% baseline, n_i=120)	1.000	.990	.635
Pooled (10% baseline, n_i=240)	1.000	1.000	.846

Note: Power for different hypothetical differences (delta) in the data (a 40-percentage point difference was reported in the original study). Case numbers per cell are rounded. Baseline: Proportion of subjects who comply with the confederate’s request in the control condition.

Study 3 (Charity donations)

Stop

Statistical power 1-β for Fisher's exact test

	Delta 40	Delta 20	Delta 10
Shirt (74% baseline, n_i=160)	1.000	.999	.544
Cap (47% baseline, n_i=140)	1.000	.904	.338
Pooled (61% baseline, n_i=290)	1.0000	1.000	.692

Note: Baseline: Proportion of subjects who stop in the control condition.

Average donation

Statistical power 1-β for t-test

		Assuming twice as high donations in brand conditions as in control, same standard error
Shirt (n_i=160)	C-L<br>C-G	d=.221, 1-β=.506<br>d=.285, 1-β=.719
Cap (n_i=140)	C-L<br>C-G	d=.253, 1-β=.533<br>d=.221, 1-β=.449
Pooled (n_i=290)	C-L<br>C-G	d=.238, 1-β=.816<br>d=.259 , 1-β=.875

Note: An approximately twice as high average donation in the experimental treatment than in the control treatment was reported in the original study. Measure of effect size: Cohen's d. Case numbers per cell are rounded.

Study 4 (Compliance, low status neighborhood)

Statistical power 1-β for Fisher's exact test

	Delta 40	Delta 20	Delta 10
Shirt (26% baseline, n_i=120)	1.000	.880	.337
Cap (44% baseline, n_i=120)	1.000	.845	.282
Pooled (35% baseline, n_i=240)	1.000	.995	.737

Note: Power for different hypothetical differences (delta) in the data (a 40 percentage point difference was reported in the original study). Case numbers per cell are rounded. Baseline: Proportion of subjects who comply with the confederate's request in the control condition.

Study 5 (Charity donations, low status neighborhood)

Stop

Statistical power 1-β for Fisher's exact test

	Delta 40	Delta 20	Delta 10
Shirt (32% baseline, n_i=120)	1.000	.861	.319

Note: Baseline: Proportion of subjects who stop in the control condition.

Average donation

Statistical power 1-β for Fisher's exact test

		Assume twice as high donations in brand conditions as in control, same standard error
Shirt (n_i=160)	C-L<br>C-G	d=.444, 1-β=.928<br>d=.353, 1-β=.777

Note: An approximately twice as high average donation in the experimental treatment than in the control treatment was reported in the original study. Measure of effect size: Cohen's d. Case numbers per cell are rounded.

Pooled: average neighborhood

Positive reaction

Statistical power (1-β) for Fisher's exact test

	Delta 40	Delta 20	Delta 10
Shirt (48% baseline, n_i=280)	1.000	.998	.620
Cap (29% baseline, n_i=250)	1.000	.995	.624
Pooled (39% baseline, n_i=530)	1.000	1.000	.899

Note: Baseline: Proportion of subjects who react positively in the control condition.

Pooled: low socio-economic status neighborhood

Positive reaction

Statistical power (1-β) for Fisher's exact test

	Delta 40	Delta 20	Delta 10
Shirt (32% baseline, n_i=120)	1.000	.861	.319
Cap (44% baseline, n_i=120)	1.000	.845	.282
Pooled (38% baseline, n_i=240)	1.00	.991	.559

Note: Baseline: Proportion of subjects who react positively in the control condition.

Pooled: all

Positive reaction

Statistical power (1-β) for Fisher's exact test

	Delta 40	Delta 20	Delta 10
Shirt (39% baseline, n_i=520)	1.00	1.00	.890
Cap (34% baseline, n_i=375)	1.00	1.00	.779
Pooled (37% baseline, n_i=890)	1.00	1.00	.989

Note: Baseline: Proportion of subjects who react positively in the control condition.
