# Supplementary material for: Are Luxury Brand Labels and “Green” Labels Costly Signals of Social Status? An Extended Replication
Source: PLoS One. 2017 Feb 7;12(2):e0170216. doi: 10.1371/journal.pone.0170216 (PMC5295666; doi:10.1371/journal.pone.0170216)
Supplement: S7 File — (PDF) [file pone.0170216.s007.pdf]

S7 File
Gender specific cross tables: Frequencies and test statistics

Legend. C: Control treatment, L: Luxury label treatment, G: Green label treatment, Exact: Fisher's exact test

Compliance (studies 2 and 4)

Female experimenter, female subject

	Control		Luxury label		Green label	
	n	%	n	%	n	%
Does not comply	137	91.33	141	90.97	119	88.15
Complies	13	8.67	14	9.03	16	11.85
Total	150	100	155	100	135	100

Overall: χ^2=.966, p=.617
CL: χ^2=.013, p=.911, exact=1.00
CG: χ^2=.789, p=.374, exact=.435
LG: χ^2= .619, p=.432, exact=.447

Female experimenter, male subject

	Control		Luxury label		Green label	
	n	%	n	%	n	%
Does not comply	70	86.42	78	82.98	89	84.76
Complies	11	13.58	16	17.02	16	15.24
Total	81	100	94	100	105	100

Overall: χ^2=.398, p=.819
CL: χ^2=.395, p=.530, exact= .675
CG: χ^2=.103 , p=.750, exact=.835
LG: χ^2= .117, p=.732, exact=.847

Male experimenter, male subject

	Control		Luxury label		Green label	
	n	%	n	%	n	%
Does not comply	25	52.08	35	63.64	35	62.50
Complies	23	47.92	20	36.36	21	37.50
Total	48	100	55	100	56	100

Overall: χ^2=1.694, p=.429
CL: χ^2=1.407, p=.236, exact= .317
CG: χ^2= 1.149, p= .284, exact=.323
LG: χ^2= .015, p= .901, exact=1.000

Male experimenter, female subject

	Control		Luxury label		Green label	
	n	%	n	%	n	%
Does not comply	45	58.44	45	69.23	38	64.41
Complies	32	41.56	20	30.77	21	35.59
Total	77	100	65	100	59	100

Overall: χ^2=1.793, p=.408

CL: χ^2=.1.768, p=.184, exact= .222
CG: χ^2=.500, p=.480, exact=.595
LG: χ^2= .325, p=.569, exact=.703

Donations (studies 3 and 5)

Female experimenter, female subject

	Control	Luxury	Green
Mean donation	. 707	.955	.566
SD	3.195	3.189	2.605
n	178	174	191

CL: |t|=.725, p=.469
CG: |t|= .467, p=.641
GL: |t|= 1.279, p=.202

Female experimenter, male subject

	Control	Luxury	Green
Mean donation	.617	.415	.491
SD	2.083	2.206	1.886
n	121	110	106

CL: |t|=.712, p=.477
CG: |t|=.475, p=.635
GL: |t|=.269, p=.789

Male experimenter, male subject

	Control	Luxury	Green
Mean donation	1.308	.388	1.065
SD	2.840	1.281	2.058
n	53	58	57

CL: |t|=2.231., p=.028
CG: |t|=.516, p=.607
GL: |t|= 2.122, p=.036

Male experimenter, female subject

	Control	Luxury	Green
Mean donation	.597	.536	1.194
SD	1.962	1.907	3.266
n	67	62	63

CL: |t|=.178, p=. 859
CG: |t|=1.271, p=.206
GL: |t|= 1.372, p=.173

Positive reaction (pooled)

Female experimenter, female subject

	Control		Luxury label		Green label	
	n	%	n	%	n	%
Negative reaction	210	64.02	208	63.22	193	59.20
Positive reaction	118	35.98	121	36.78	133	40.80
Total	328	100	329	100	326	100

Overall: χ^2=1.855, p=.396
CL: χ^2=.046, p=.831, exact= .871
CG: χ^2= 1.607, p=.205, exact=.228
LG: χ^2= 1.114, p=.291, exact=.298

Female experimenter, male subject

	Control		Luxury label		Green label	
	n	%	n	%	n	%
Negative reaction	113	55.94	121	59.31	130	61.61
Positive reaction	89	44.06	83	40.69	81	38.39
Total	202	100	204	100	211	100

Overall: χ^2=1.385, p=.500
CL: χ^2=.473, p=.492, exact= .547
CG: χ^2=1.370, p=.242, exact=.271
LG: χ^2= 0.229., p=.632, exact=.688

Male experimenter, male subject

	Control		Luxury label		Green label	
	n	%	n	%	n	%
Negative reaction	60	59.41	77	68.14	75	66.37
Positive reaction	41	40.59	36	31.86	38	33.63
Total	101	100	113	100	113	100

Overall: χ^2=1.966, p=.374
CL: χ^2=1.767, p=.184, exact= .201
CG: χ^2=1.111, p=.292, exact=.322
LG: χ^2= 0.080, p=.777, exact=.887

Male experimenter, female subject

	Control		Luxury label		Green label	
	n	%	n	%	n	%
Negative reaction	92	63.89	91	71.65	80	65.57
Positive reaction	52	36.11	36	28.35	42	34.43
Total	144	100	127	100	122	100

Overall: χ^2= 1.983, p=.371
CL: χ^2= 1.855, p=.173, exact= .195
CG: χ^2=.082, p=.775, exact=.798
LG: χ^2=1.069 , p=.301, exact=.340
