# Supplementary material for: Are Luxury Brand Labels and “Green” Labels Costly Signals of Social Status? An Extended Replication
Source: PLoS One. 2017 Feb 7;12(2):e0170216. doi: 10.1371/journal.pone.0170216 (PMC5295666; doi:10.1371/journal.pone.0170216)
Supplement: S8 File — (PDF) [file pone.0170216.s008.pdf]

S8 File
Logit regression models with and without study-specific dummy variables

Average status neighborhood

	Positive reaction		Positive reaction	
	Coeff.	s.e.	Coeff.	s.e.
Control	*Ref.*		*Ref.*	
Luxury	-.033	.126	.043	.146
Green	.033	.125	.075	.145
Study dummies	*no*		*yes*	

Low status neighborhood

	Positive reaction		Positive reaction	
	Coeff.	s.e.	Coeff.	s.e.
Control	*Ref.*		*Ref.*	
Luxury	-.356	.193†	-.355	.193†
Green	-.170	.190	-.163	.191
Study dummies	*no*		*yes*	

Note: Coefficients and standard errors from logit regression models. $^{\dagger}p<.10$, $^{*}p<.05$.

Pooled

	Positive reaction		Positive reaction	
	Coeff.	s.e.	Coeff.	s.e.
Control	*Ref.*		*Ref.*	
Luxury	-.099	.099	-.070	.108
Green	.015	.098	.034	.107
Study dummies	*no*		*yes*	

Note: Coefficients and standard errors from logit regression models. $^{\dagger}p<.10$, $^{*}p<.05$.

Pooled: average neighborhood

Shirt

	Control		Luxury label		Green label	
	n	%	n	%	n	%
Negative reaction	145	51.79	146	52.14	152	54.29
Positive reaction	135	48.21	134	47.86	128	45.71
Total	280	100	280	100	280	100

Overall: χ^2= .410, p= .814
CL: χ^2= .007, p= .933, exact=1.000
CG: χ^2=.351, p= .553, exact=.611
LG: χ^2= .611, p= .258, exact=.672

Cap

	Control		Luxury label		Green label	
	n	%	n	%	n	%
Negative reaction	178	71.20	183	72.33	171	66.54
Positive reaction	72	28.80	70	27.67	86	33.46
Total	250	100	253	100	257	100

Overall: χ^2= 2.294, p= .318
CL: χ^2= .080 , p= .778, exact=.843
CG: χ^2= 1.285, p= .257, exact=.292
LG: χ^2= 2.017, p= .156, exact=.178

Pooled

	Control		Luxury label		Green label	
	n	%	n	%	n	%
Negative reaction	323	60.94	329	61.73	323	60.15
Positive reaction	207	39.06	204	38.27	214	39.85
Total	530	100	533	100	537	100

Overall: χ^2= .280, p=.870
CL: χ^2= .069, p=.793, exact=.801
CG: χ^2=.071, p= .791, exact=.802
LG: χ^2=.280, p=.597, exact=.616

Shirt vs. cap: χ^2=52.488, p=.000, exact=.000

Pooled: low status neighborhood

Shirt

	Control		Luxury label		Green label	
	n	%	n	%	n	%
Negative reaction	82	68.33	88	73.33	82	68.33
Positive reaction	38	31.67	32	26.67	38	31.67
Total	120	100	120	100	120	100

Overall: χ^2=.952 , p= .621
CL: χ^2= 0.726, p= .394 , exact= .478
CG: χ^2= .000, p= 1.000, exact= 1.000
LG: χ^2= 0.726, p= .394, exact=.621

[Cap low status study is cap study 4]

Overall

	Control		Luxury label		Green label	
	n	%	n	%	n	%
Negative reaction	152	62.04	168	70.00	155	65.96
Positive reaction	93	37.96	72	30.00	80	34.04
Total	245	100	240	100	235	100

Overall: χ^2=3.421 , p=.181
CL: χ^2= 3.421 , p= .064, exact=.069
CG: χ^2=.798, p= .372, exact=.393
LG: χ^2=0.892, p=.345, exact=.376

Shirt vs. cap: χ2= 7.912, p=.005

Pooled: all

Shirt

	Control		Luxury label		Green label	
	n	%	n	%	n	%
Negative reaction	316	60.77	320	61.54	315	60.58
Positive reaction	204	39.23	200	38.46	205	39.42
Total	520	100	520	100	520	100

Overall: χ^2= .113, p = .945
CL: χ^2= .065, p = .799, exact =.849
CG: χ^2=.004, p = .949, exact=1.000
LG: χ^2= .101, p = 751., exact=.799

Cap

	Control		Luxury label		Green label	
	n	%	n	%	n	%
Negative reaction	248	66.13	263	70.51	244	65.59
Positive reaction	127	33.87	110	29.49	128	34.14
Total	375	100	373	100	372	100

Overall: χ^2=2.469, p = .291
CL: χ^2=1.654, p = .198, exact=.209
CG: χ^2 = .024, p = .876, exact=.878
LG: χ^2= .039, p = .843, exact=.895

Overall

	Control		Luxury label		Green label	
	n	%	n	%	n	%
Negative reaction	564	63.02	583	65.29	559	62.67
Positive reaction	331	36.98	310	34.71	333	37.33
Total	895	100	893	100	892	100

Overall: χ^2=1.559, p=.459
CL: χ^2= 1.001, p =.317 , exact =.324
CG: χ^2=.023, p=.879 , exact=.883
LG: χ^2=1.327 , p=.249 , exact=.257

Shirt vs. cap χ^2= 98.555, p=.000, exact=.000
